# Gelseminum in Odontalgia

**Published:** 1873-11

**Authors:** J. W. Legg

**Affiliations:** London Lancet.


### ARTICLE VI.

### *Gelseminum in Odontalgia.*

Gelseminum sempervirens is a plant growing in the Southern States of North America. Its active properties were discovered by accident. It has been for many years past used in America, chiefly by irregular practitioners. A few months ags I happened to hear of its being employed with very great success in a case of pains in the jaw from decayed teeth, and I determined to make some inquiry into its therapeutical virtues. Mr. Jeffs, the apothecary of St. Bartholomew's Hospital, very kindly prepared for me a tincture by macerating for a week an ounce of the root with eight ounces of proof spirit. All the following observations were made with this preparation.

The cases in which I have found this drug of most use are those of bad tooth-ache, which the patients themselves, and even some practitioners, call neuralgia. Short notes of some of these cases follow.

Mary L——, seventeen years old, came March 10th, 1873, complaining of shooting pains in the right side of the face, which had lasted for nine weeks. There were many decayed teeth. She had taken citrate of iron with benefit, but the pains returned when the medicine was left off. Ordered to take ten minims of the tincture of gelseminum in water every three hours. Came on March 13th, saying that the pains had ceased completely the day following the first administration of the drug. To take now instead the quassia and iron mixture of the hospital.

Emma B——, aged twenty, came on January 6th, 1873, complaining of pain in the lower and upper jaws, lasting three weeks, and in the head. Last night had no sleep. The pains last two or three hours and then cease. Many decayed teeth in both jaws. To take fifteen minims of the tincture of gelseminum in water, every three hours.—January 9th: Pains much relieved; eating brings them on again, "it strikes cold." To continue the gelseminum.

Richard T——, aged, fifty-three, came on March 22d, 1873, complaining of shooting pains in the upper jaw since Christmas, coming on about four o'clock every day and lasting till midnight; many decayed teeth. To take twenty minims of tincture of gelseminum, in water, every three hours—March 26th; Pains ceased on the 23d of March, the day after the first visit. To take the quassia and iron mixture three times daily.

Fanny S——, aged thirty-eight, came on March 24th, 1873, complaining of pains in the face lasting for fourteen days; throbbing, paroxysmal; gums swollen; many decayed teeth. To take twenty minims of tincture of gelseminum, in water, every three hours. She came again on March 27th, saying the pains were bad all the night of the 24th, but ceased on the 25th, and had not returned. She now only feels weak. To take the quassia and iron mixture.—*J. W. Legg, M. D., in London Lancet.*

---

